# Infant feeding practices among HIV exposed infants using summary index in Sidama Zone, Southern Ethiopia: a cross sectional study

**DOI:** 10.1186/1471-2431-14-49

**Published:** 2014-02-18

**Authors:** Demewoz Haile, Tefera Belachew, Getenesh Birhanu, Tesfaye Setegn, Sibhatu Biadgilign

**Affiliations:** 1 Department of Public Health, College of Medicine and Health Sciences, Madawalabu University, P.o. Box: 139 Bale, Goba, Ethiopia; 2 Population and family Health Department, College of Public Health and Medical Sciences, Jimma University, Jimma, Ethiopia; 3 Department of applied human nutrition, School of food sciences and Nutrition, Hawassa University, Hawassa, Ethiopia; 4 Department of Reproductive Health, College of Medicine and Health Sciences, Bahir Dar University, Bahir Dar, Ethiopia; 5 Independent Public Health Consultants, Addis Ababa, Ethiopia

## Abstract

**Background:**

Combining various aspects of child feeding into an age-specific summary index provides a first answer to the question of how best to deal with recommended feeding practices in the context of HIV pandemic. The objective of this study is to assess feeding practices of HIV exposed infants using summary index and its association with nutritional status in Southern Ethiopia.

**Methods:**

Facility based cross-sectional study design with cluster random sampling technique was conducted in Sidama Zone, Southern Ethiopia. Bivariate and multivariable linear regression analyses were performed to assess the association between summary index (infant and child feeding index) (CS-ICFI) and nutritional status.

**Results:**

The mean (±standard deviation (SD)) cross-sectional infant and child feeding index (CS-ICFI) score of infants was 9.09 (±2.59), [95% CI: 8.69-9.49]). Thirty seven percent (36.6%) of HIV exposed infants fell in the high CS-ICFI category while 31.4% of them were found in poor feeding index tertile. About forty two percent (41.6%) of urban infants were found in the high index tertile but only 24% of the rural infants were found in high index tertile. Forty six percent (46%) of the rural infants were found in low (poor) feeding index category. The CS-ICFI has a statistically significant association with weight for age z score (WAZ) (ß = 0.168, p = 0.027) and length for age z score (LAZ) (ß = 0.183 p = 0.036). However CS-ICFI was not significantly associated with weight for height z score (WLZ) (p = 0.386).

**Conclusion:**

Majority of HIV exposed infants had no optimum complementary feeding practices according to cross-sectional infant and child feeding index. CS-ICFI was statistically associated especially with chronic indicators of nutritional status (LAZ and WAZ). More rural infants were found in poor index tertile than urban infants. This may suggest that rural infants need more attention than urban infants while designing and implementing complementary feeding interventions.

## Background

The dilemma posed by Human Immunodeficiency Virus (HIV) pandemic and the risk of mother to child transmission (MTCT) of HIV especially during breast feeding has been a challenge to public health interventions at large [1-3]. Although World Health Organization/ United Nation Children Fund (WHO/UNICEF) has recommended two years of continuous breastfeeding, children born from HIV positive mothers have not been benefited from this recommendation due to the risk of mother to child transmission of the virus. Appropriate infant and young child feeding practices in the context of HIV should balance the risk of mother to child transmission of the virus and morbidity and mortality from other causes. The new WHO guideline on HIV and infant feeding practices recommended that HIV-infected mothers whose infants are HIV negative or of unknown status to breastfeed exclusively for the first 6 months, then introduce complementary foods and continue to breastfeed for the first 12 months of life [4]. The Ethiopian national guideline for prevention of mother to child transmission (PMTCT) recommends that breast feeding for HIV exposed infants should be continued at least for 12 months and at most for 18 months [5].

It is evidenced that malnutrition rate increases during the period of transition from exclusive breast feeding to complementary feeding which might be partly due to inappropriate feeding practices [6]. In fact weight and height gain during infancy are influenced by infant feeding practices [7-9]. The intersecting effect of inappropriate feeding practices among HIV exposed infants followed by malnutrition has been resulted in significant increase in child mortality after 6 months of age [10]. However infant and young child practices are multidimensional and dynamic within short age intervals. Hence, measuring feeding practices of infants and young children greater than 6 months of age is complex [11-13].

Although the challenge has persisted, considerable progress has been made in defining standards and indicators for appropriate complementary feeding practices through the development of indicators for assessing infant and young child feeding practices [14]. The previously developed indicators could not able to show the simultaneous effect of different dimension of complementary feeding and has focused on single practices over a narrow age range and has not addressed the impact of adequate or optimal infant and child feeding [15]. Therefore developing an index which able to reflect both feeding behavior and diet quality in measuring feeding practices of infants greater than 6 months of age at a time is important [16]. Besides to this, quantifiable summary index increases the comparability of findings of different studies in the area of child feeding [13,15].

So far infant and child feeding index (ICFI) has only been used for non HIV exposed children. The application of this summary index for assessing feeding practices of HIV exposed infants is not known in Ethiopia by considering the current feeding recommendations for HIV exposed infants.

Therefore, the objective of this study is to assess the infant feeding practices of HIV exposed infants using summary index and its association with their nutritional status in Sidama zone, Southern Ethiopia which would provide policy makers, program implementers and care providers with evidence-based information on optimal complementary feeding practices of HIV exposed infants.

## Methods

### Study setting and sample

This study was conducted in Sidama zone which is one of the zones in South Nation, Nationalities and People Regional state (SNNPR) of Ethiopia. Fifty percent (50.48%) of the total population was male while 49.51% were female [17]. A substantial area of Sidama land produces coffee, which is the major cash crop in the region, and larger number of the population is known to heavily depend on ‘*Enset*’ (false Banana). The staple foods in Sidama Zone are maize and *kocho*[18]. Kocho is bulky, chewy, fermented starch bread which is made from a mixture of the decorticated leaf sheaths and grated root.

A facility-based cross-sectional study was conducted in randomly selected 10 government health institutions which were providing ART (antiretroviral therapy) and PMTCT (prevention of mother to child transmission) services in Sidama Zone, Southern Ethiopia between February and April 2012. There were 18 health institutions which were providing ART and PMTCT services for HIV positive mothers with their HIV exposed infants. From the total of 18 health institutions, four were excluded based on the exclusion criteria and the remaining fourteen health institutions were considered as cluster and ten health institutions (clusters) were selected randomly. All (n = 184) HIV exposed infant-mother pairs from randomly selected health institutions were included in the study. Those mothers who have HIV exposed infant of aged 6–17 months and absence of serious illness of the mother or infant were the inclusion criteria’s. Infants were excluded from the study if they were not exposed to HIV or if they were diagnosed as HIV-positive prior to data collection. None of the mothers who fulfill the eligibility criteria were found to refuse to participate in the study. A pre-tested structured questionnaire was used to collect socio-demographic and feeding practices of HIV exposed infant. Feeding practices were assessed by the qualitative 24 recall method and 7 day quasi food group frequency. Health professionals were recruited and trained for two days on data collection techniques. The data collection process was closely supervised and collected data were checked for completeness and inconsistencies in the field.

### Anthropometric measurements

All anthropometric measurements were taken by trained nurses with their respective assistants. Length of the infants (6–17 months) was measured in a recumbent position to the nearest 0.1 cm using a locally made wooden sliding board with an upright wooden base and movable headpiece. Weight was measured in kilogram to the nearest 0.1 Kg by Salter hanging scale. Calibration of instrument against zero reading was checked after weighting every infant. Instruments were checked against a standard weight for its accuracy daily. Infants were weighed with light clothing and without shoes.

### Cross-sectional infant and child feeding index (CS-ICFI)

The CS-ICFI were constructed using the method proposed by Ruel and Menon [15] and adapted to the local context and to the current recommendation (Table 1). The 24 hour dietary diversity score is a sum score of: Grains + Tubers + Milk + Vitamin A-rich fruits/vegetables + other fruits/vegetables/juice + Animal source foods + Legumes + Fats (received, or did not receive each food/group). Scores were assigned to reflect the age-specific distributions of HIV exposed infants in tertiles. The seven day quasi food frequency is a modified food group frequency and measured as "How many days in the last seven days was given [food group]?" The number of days that a food group has consumed recorded for each child with a maximum of seven days.

**Table 1:** Feeding practices and scoring system used to create infant/child feeding index for HIV exposed infants aged 6–17 months, by age group, 2012

**Variables**	**6**-**9 months**	**9** -**11 months**	**12** -**17 months**
Current breast feeding	No = 0	No = 0	No = 1
	Yes = 2	Yes = 2	Yes =1
Bottle feeding (24 Hrs recall)	Yes =0	Ye =0	Yes =0
	No = 1	No = 1	No = 1
Dietary diversity (past 24 hours)	None of the foods/groups: Score = 0	None of the foods groups :Score = 0	None or one of the foods/groups: Score = 0
	One food group: score = 1	One to two foods groups: Score = 1	Two or three foods/groups: Score = 1
	Two or more food groups: score = 2	Three or more food groups: Score = 2	Four or more foods/groups: Score = 2
Frequency of feeding	Not at all: Score = 0	Not at all: Score = 0	Not at all or once: Score = 0
solids/semi-solids (past 24 hours)	Once: Score = 1	Once or twice: Score = 1	Twice: Score = 1
	2 or more times: Score = 2	3 or more times: Score = 2	Three times: Score = 2
			Four times or more: Score = 3
Seven day qusi food group frequency ^@^	0 (no foods prev. week): Score = 0	0 or 1: Score = 0	0 through 3: Score = 0
	1 or 2: Score = 1	2 through 4: Score = 1	4 through 6: Score =1
	3 or higher: Score =2	5 or higher: Score = 2	7 or higher: Score =2
Mother reports hand washing before cooking food	Yes =1	Yes =1	Yes =1
	No =0	No =0	No =0
Wash hand before feeding the child	Yes =1	Yes =1	Yes =1
	No = 0	No = 0	No = 0
Does the infant get help to eat yesterday?	Yes = 1	Yes = 1	Yes = 1
	No =0	No =0	No =0
What does caregiver do when child refuses to eat?			
A) Nothing (child left alone)	a = 0	a = 0	a = 0
B) Other *	b = 1	b = 1	b = 1
Total (maximum , minimum)	13,0	13,0	13,0

*coax, play with, force, change food, not a problem., @ This is a modified food group frequency, where the questions are asked in the form, How many days in the last seven days was (name) given (food group)?,î so that the number entered for each child is the number of days, with a maximum of seven, not the number of times the child ate a food from the group.

The list of foods summed is the same as for the 24-hour diversity score, with the exception that grains have been combined with roots/tubers. In seven day food group frequency score, each food group is scored 0 if not given to the infant in the previous week, scored +1 if given one to three days, and +2 if given four or more days in the previous week. These scores are then summed to give a possible range of 0 to 14 and the seven days food group frequency scores were assigned to reflect the age-specific distributions of study participants in tertiles. The CS-ICFI was developed with values 0–13 and it was divided into 3 categories (tertiles) in the following manner: a sum scores of 0–7 categorized as low CS-ICFI, sum scores of 8–10 categorized as medium CS-ICFI, and sum scores of 11–13 were classified as high CS-ICFI.

### Statistical analysis

Data were checked for completeness, consistencies, cleaned, coded and entered to SPSS for windows version 20.0. It was exported to WHO Anthro for nutritional status analysis. Descriptive statistics (mean, standard deviation, minimum, maximum, and median) were computed for all continuous variables and frequency distribution was carried out to evaluate the distribution of categorical variables. Homogeneity of variance and normality assumption were tested and found that the data was fit for ANOVA analysis and independent *t*-test. Bivariate analyses (independent *t*-test or one way ANOVA) were carried out at the first stage. Then multivariable linear regression model was fitted to identify independent association between CS-ICFI and nutritional status of HIV exposed infants. All tests were two-sided and p < 0.05 was considered for statistical significance. The internal consistency of the CS-ICFI was measured by the Cronbach’s α coefficient. The Cronbach’s α value higher than 0.7 was generally considered to be satisfactory [19]. The anthropometric indices were computed and compared with reference data from World Health Organization growth chart 2007. Children below-2SD of the WHO median weight-for-age, height-for-age and weight-for-height were considered as underweight, stunted and wasted, respectively.

Ethical approval was received from institutional review board (IRB) of Hawassa University. Official letter of cooperation was also obtained from Sidama Zone Health Department. Informed verbal consent was secured from study participants in their own language after explaining the purpose of the study, potential risk and benefits of participating in the study. The right of respondents to withdraw from the study any time was assured. The participants were also assured about the confidentiality of the data.

## Result

### Socio-demographic characteristics

A total of 184 HIV positive mothers having HIV exposed infants of aged 6–17 months were included in the study. The mean (±SD) age of mothers was 28.85 (±5.4) years. About fifty five percent (54.9%) of the respondents were protestant by religion and 77 (43.03%) were illiterates by educational status. Majority 158 (84.5%) were married and 134 (72.8%) of the respondents were urban residents (Table 2).

**Table 2:** **Socio**-**demographic characteristics of mothers having HIV exposed infants in Sidama Zone**, **Southern Ethiopia**, **2012**

***Socio****-****demographic characteristics***	***Frequency***	***Percentage***
Age of the mother(years)(n = 179)		
≤24	28	15.6
25-29	73	40.8
≥30	78	43.6
Religion(n = 184)		
Protestant	101	54.9
Orthodox	64	34.8
Muslim	15	8.2
Catholic	4	2.2
Ethnic group of mothers(n = 184)		
Sidama	62	33.7
Amhara	37	20.1
Gurage	30	16.3
Oromo	28	15.2
Wolayita	17	9.2
Others *	10	5.4
Marital status(n = 181)		
Married	158	84.5
Widowed	12	6.5
Divorced	11	7.61
Place of residence(n = 184)		
Urban	134	72.8
Rural	50	27.2
Educational status(n = 179)		
Illiterate^ε^	77	43.03
Read /write	8	4.47
Primary education (1–8)	53	29.61
Secondary education (9^+^)	41	22.92
Sex of infant (n = 184)		
Male	106	57.6
Female	78	42.4

^*ε*^those who didn’t attend any formal education *Tigre, Kambata and Gamo.

### Cross sectional infant and child feeding index score of infants (CS-ICFI)

**Figure 1:**
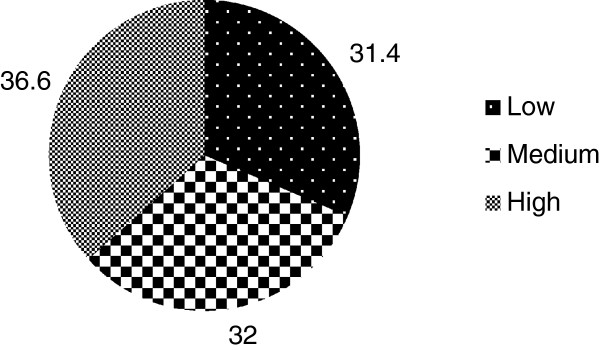
**Distribution of HIV exposed infants by their feeding index tertile in Sidama Zone**, **South Ethiopia**, **2012**.

The mean (±SD) cross sectional infant and child feeding index (CS-ICFI) score of infants was 9.09 (±2.59), [95% CI: 8.69-9.49]. The mean (±SD) CS-ICFI scores for respective age groups were 8.19 (±2.71), [95% CI: 7.58- 8.85], 9.33 (±2.37), [95% CI: 8.57-10.09] and 9.85 (±2.39), [95% CI: 9.22-10.35] for infants 6–8 months of age, 9–11 months of age and 12–17 months of age respectively. There was a statistically significant difference in mean CS-ICFI among those age groups (p = 0.002). The infant and child feeding index score varied from a minimum of 3 to a maximum of 13 (for a theoretical maximum of 13). About thirty seven percent (36.6%) of infants were under high CS-ICFI tertile while (31.4%) of them were found in the low CS-ICFI tertile (Figure 1).

**Figure 2:**
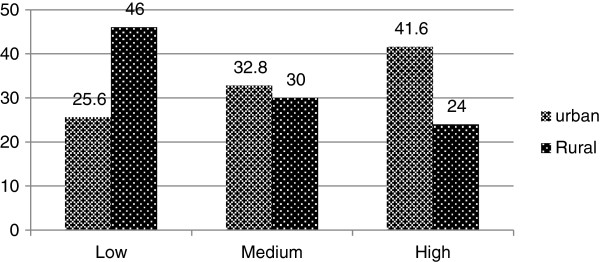
**Distribution of CS**-**ICFI tertiles by place of residence among HIV exposed infants in Sidama Zone**, **South Ethiopia**, **2012**.

There was a statistically significant difference in CS-ICFI mean scores of urban and rural infants (9.34 Vs 8.44) (p = 0.037). About 41.6% of urban infants were found in the high index tertile but only 24% of the rural infants were found in high index tertile. The prevalence of low (poor) feeding index tertile was 46% in the rural infants (Figure 2). ART status (pre-ART or on ART) and disclosure of HIV status were not statistically associated with CS-ICFI tertiles (p > 0.05). But the time when the mother know their sero status has statistically significant association with CS-ICFI category (p = 0.022) (Table 3).

**Table 3:** **Association of CS**-**ICFI of HIV exposed infants and maternal characteristics in Sidama Zone**, **South Ethiopia**, **2012**

**Characteristics**		**CS**-**ICFI tertiles**						***P value***
		**Low**		**Medium**		**High**		
		**CS**-**ICFI**		**CS**-**ICFI**		**CS**-**ICFI**		
		***No***	***%***	***No***	***%***	***No***	**%**	
ART status	Pre ART	24	43.6	15	27.3	18	28.1	0.115
	On ART	31	56.4	40	72.7	46	71.9	
Disclosure of HIV status	Yes	47	85.5	49	87.5	55	85.9	0.947
	No	8	14.5	7	12.5	9	14.1	
Stigma and discrimination	Yes	3	5.5	8	14.3	4	6.2	0.200
	No	53	94.5	48	85.7	60	93.8	
	No	48	87.3	42	75	30	47.6	
When you know your HIV status	Before pregnancy	21	38.2	32	57.1	35	54.7	0.022*
	During pregnancy	30	54.5	16	28.6	18	28.1	
	During birth	2	3.6	1	1.8	1	1.6	
	After birth	2	3.6	7	12.5	10	15.6	

*significant p value <0.05.

### Evaluation of the internal consistency of CS-ICFI

The internal consistency of the index was estimated by the Cronbach’s α coefficient [19]. The Cronbach’s α coefficient of this study was more or less similar among the different age groups. But the Cronbach’s α value was slightly higher for older age groups. The CS- ICFI internal consistency was good for infants aged 9–11 months α = 0.70 (95% CI: 0.49-0.80) and aged 12–17 months α = 0.71 (95% CI: 0.55-0.78), and it was lower for infants aged 6–8 months α =0.68 (95% CI: 0.54-0.77).

The CS-ICFI showed strong correlation with 24 hour food frequency score, 24 hour food diversity score and seven day food group frequency. The correlation between current breast feeding score and CS-ICFI decreased in the older age groups. In youngest and oldest age groups, removing breast-feeding and bottle-feeding from the index improved the value of Cronbach’s α coefficient to the acceptable range (≥0.70). For all age groups removing the bottle feeding from the index improved the value of the Cronbach’s α coefficient to the acceptable range (≥0.70) (Table 4).

**Table 4:** **Internal consistency of CS**-**ICFI and its correlation with ICFI components among HIV exposed infants in Sidama Zone**, **South Ethiopia**, **2012**

	**All (n = 175)**		**6-8 months (n = 69)**		**9**-**11 months**		**12**-**17**	
					**(n = 44)**		**months (n = 62)**	
Cronbach’s α^¢^	0.67		0.68		0.70		0.71	
Components of ICFI	r*	α‟	r*	α‟	r*	α‟	r*	α‟
Meal frequency score^#^	0.81	0.56	0.77	0.55	0.68	0.6	0.78	0.59
Dietary diversity score^#^	0.80	0.59	0.69	0.55	0.61	0.61	0.73	0.62
7 day food frequency	0.71	0.6	0.53	0.56	0.4	0.64	0.61	0.64
Psycho social support during food refusal^#^	0.32	0.67	0.33	0.68	0.27	0.67	.0.50	0.67
Infant get help to eat^#^	0.65	0.65	0.44	0.65	0.35	0.65	0.56	0.66
Bottle feeding^#^	-0.15	0.71	0.41	0.72	-0.06	0.70	0.10	0.72
Washing hands before cooking child food^#^	0.67	0.67	0.29	0.67	0.20	0.67	0.24	0.68
Wash hands before feeding ^#^	0.67	0.67	0.28	0.55	0.23	0.67	0.39	0.69
Current breast feeding^#^	0.02	0.71	0.46	0.72	0.27	0.66	0.27	0.70

^¢^α value when all items included *correlation of each component with CS-ICFI ‟α value when Item removed, ^#^24 hour recall.

### Association between nutritional status and CS-ICFI

Forty two (23.7%) of the HIV exposed infants were stunted and 27 (15.3%) were underweight while 23 (13.5%) were wasted. The mean WLZ, LAZ and WAZ was -0.19, -0.86, and -0.72, respectively.

The bivariate analysis showed that the mean LAZ and WAZ score of HIV exposed were statistically different across with CS-ICFI tertiles (Table 5). Multivariable linear regression analysis was also performed to determine the statistical association between CS-ICFI and WAZ after controlling the effect of potential confounders. After adjusting for diarrheal morbidity in the last two weeks, WAZ score was significantly associated with CS-ICFI (ß = 0.168, p = 0.027). The association between CS-ICFI and LAZ scores was statistically significant. Monthly income (positively) (p = 0.022) and pre-lacteal feeding (negatively) (p = 0.048) were independent predictors of LAZ score (Table 6).

**Table 5:** **Bivariate association** (**one way ANOVA**) **of CS**-**ICFI and nutritional status among HIV exposed infants in Sidama Zone**, **South Ethiopia**, **2012**

**Nutritional status**	**CS**-**ICFI**			**F value**	**P value**
	**Low**	**Medium**	**High**		
Mean WLZ^**^**^	0.12	-0.29	-0.28	0.957	0.386
Mean WAZ†	-1.12	-0.60	-0.43	3.611	0.029&
Mean LAZ^✦^	-1.70	-0.54	-0.31	3.621	0.029&

^**^**^weight for length Z score^†^weight for age Z score ^✦^length for age Z score &statistically significant at p < 0.05(two-tailed).

**Table 6:** **Multivariable linear regressions to identify association between LAZ score and CS**-**ICFI among HIV exposed infants in Sidama Zone**, **South Ethiopia**, **2012**

**Variables**	**LAZ**	
	**ß**	**P value**
Marital status(married)	0.054	0.505
Monthly income	0.199	0.022^†^
Pre lacteal feeding	-0.162	0.048^†^
Age at introduction of CF	-0.062	0.501
CS-ICFI	0.183	0.036^†^

ß *= standardized regression coefficients* CF *= Complementary Feeding*^*†*^*statistically significant at p < 0.05(two-tailed).*

The interaction effects of child, maternal or household characteristics on the association between ICFI tertiles and infant nutritional status was checked by interaction model. None of those characteristics have interaction effect on the association between CS-ICFI and nutritional status (LAZ and WAZ scores) of HIV exposed infants.

## Discussion

This study has assessed feeding practices of HIV exposed infants using a cross sectional summary index which summarize key complementary feeding practices in to a composite index by considering the current infant feeding recommendations. The mean (±SD) CS-ICFI score was 9.09 (±2.59) (95% CI: 8.69-9.49). This finding is significantly higher than the mean score reported in Rwanda which is 8.04 [20]. The difference might be due to the difference in age category of study subjects. The study done in Rwanda included younger infants (6–15 months) as compared to the current study (6–17 months). The mean CS-ICFI score of the youngest age group infants was significantly different from mean CS-ICFI score of oldest age group (p = 0.002). Other studies also reported that the older age groups had higher mean index score [21,22]. This implies that complementary feeding practices among HIV exposed infants around initiation of complementary feeding practices are less optimal as compared with complementary feeding practice on the older age time.

There was significant difference in CS-ICFI mean scores between urban and rural infants (9.34 Vs 8.44) (p = 0.02). Similar finding was reported from China in which the mean CS-ICFI scores between urban and rural infants were significantly different (p < 0.05) [12,23]. The internal consistency of the index for the whole sample was a little below the acceptable limit (0.67 Vs 0.70) [19]. But removing either bottle feeding or breast feeding from the index increased the Cronbach’s α coefficient to the acceptable range. This indicated that breast feeding and bottle feeding had weak or negative correlation with other complementary dimensions. This again implicate that bottle feeding and breast feeding practice displace other complementary feeding practices. This finding is consistent with the study done in rural Senegal that omitting breast feeding component from the index increase value to 0.82 [24]. In the current study the internal consistency of CS- ICFI was good for children aged 9–11 months (α = 0.70) and for those aged 12–17 months (α = 0.71), but it was lower for infants aged 6–8 months (α =0.68). This showed that CS-ICFI is a reliable measure of complementary feeding practices for infants aged 9 months and above. The lower internal consistency of the CS-ICFI for infants of age 6–8 months resulted from higher prevalence of breast feeding practices in this age group as compared to older age groups (p = 0.003). However, this finding is not consistence with the finding from rural Burkina Faso which showed that the internal consistency was good for the youngest infants (6–11 months) (α = 0.79) and lower among children aged 12–23 months (Cronbach’s α = 0.63) [25]. This contradiction might be due to the difference in the practices of breast feeding and the socio cultural difference between the two communities. Breast-feeding was almost ubiquitous and was prolonged (even above two years) in Burkina Faso.

In this study, 36.6% of HIV exposed infants were in high CS-ICFI tertile while 31.4% of them were in lower CS-ICFI tertile. However, the age specific ICFI analysis showed that most of the infants (48.4%) in the youngest age group (6–8 months) were found in the lower CS-ICFI category while most (48.5%) of the infants in the oldest age groups (12–17 months) found in the medium CS-ICFI tertile. This finding is consistent with a study done in rural Burkina Faso which showed that among infants of aged 6–11 months, 52%, 23% and 25% were found in the poor, average and good feeding index while among children of aged 12–23 months 35%, 37%, 28% were found in poor, medium and good feeding index category respectively [25].

This study showed that there was a statistically significant difference in mean LAZ and WAZ even after controlling the potential confounders. A similar finding was reported from India which revealed that LAZ and WAZ had showed significant association with ICFI [13]. Another study from India showed that complementary feeding index was associated with LAZ score but not with WAZ and WLZ scores [21]. On the other hand a study conducted in Bangladesh reported that the mean LAZ score of children aged 12–23 months was significantly higher among those who were at the upper ICFI tertile compared to those who were at the middle or lower ICFI tertile (-2.01 and -3.20 respectively) [26]. A similar study done in Rwanda indicated that ICFI was positively associated with WLZ and WAZ scores. However, neither the ICFI nor any of its components were associated with the LAZ score [27]. There was also a study from rural Senegal reported that feeding index was not associated with either height-for-age or with linear growth [28]. But a study from China indicated that ICFI was associated with both WAZ and WLZ scores, and did not show statistically significant association with children’s LAZ score [22] which could be due to the differences in the participants’ age group in which the study participants in China were infants of aged 6–11 months. This justification is further supported by a study conducted in Latin America which conclude that the association between feeding practices and HAZ score of children was generally weaker and less consistent among children in 12 months of life but increased gradually with age [29]. The Latin American study explained the observed statistical association between feeding practices and LAZ in older infants as compared to their younger infants could be explained by the clustering/cumulative effects of previous feeding practices. In the current study the difference in mean WAZ and LAZ scores between the lowest and the highest tertile was 0.69 and 1.39 respectively. This mean difference in Z score between the two extreme CS-ICFI tertiles was statistically significant and biologically important for both WAZ and LAZ [30]. In this study, WLZ score was not associated with CS-ICFI which is consistent with other studies [21,26].

Presence of statistically significant association between CS-ICFI and WAZ and LAZ but not with WLZ may implicate that CS-CFI has the ability to reflect chronic malnutrition among infants. Thus the CS-ICFI summarizes information on feeding practices and can be used to illustrate the strength and magnitude of associations between adequate complementary feeding practices and infant nutritional outcomes in long term.

None of the two-way interactions between the CS-ICFI and the child, maternal, and household characteristics included in the model were statistically significant. Thus, it appears that the magnitude of differences in LAZ and WAZ between feeding tertiles was not conditioned by any of the child, maternal, and household factors. But a Chinese study showed that better feeding practices were more important for children of lower socioeconomic status [12]. Finally the findings of this study support the existing literature despite the fact that the index constructed in this study include hygiene and psychosocial variables as index component. The index is applicable for measuring complementary feeding practices comprehensively which include both feeding behavior and diet quality among HIV exposed infants. However further study is recommended on best approaches of assessing hygiene and psychosocial practices during complementary feeding practices.

### Limitation of the study

Interpersonal measurement error, recall bias, and absence of validated questionnaire to assess hygiene and psychosocial care were the possible limitations of this study. There might be also bias that could be introduced by the data collectors. Equal weights during scoring were given for all feeding practices especially who have binary responses. But the actual effect of those feeding practices on nutritional status might not be similar. Since the study used cross-sectional design so that it is difficult to establish cause and effect relationship between nutritional status and summary index.

## Conclusion

Majority of HIV exposed infants had no optimum complementary feeding practices. The CS-ICFI can consistently measure feeding practices of HIV exposed infants older than 9 months. The CS-ICFI was significantly associated especially with chronic indicators of nutritional status. The difference in WAZ and LAZ score between low and high tertiles of the index was biologically meaningful. More rural infants were found in poor index tertile than urban infants. This may suggest that rural infants need more attention than urban infants while designing and implementing complementary feeding interventions.

## Competing interests

The authors declare that they have no competing interests.

## Authors’ contributions

DH conceived and designed the study supervise the data collection, performed analysis, interpretation of data and drafted the manuscript. TB assisted with the design, conception, analysis, and interpretation of data and critically reviewed the manuscript. GB assisted the study design, data interpretation and critically reviewed the manuscript. TS assisted data entry, analysis, interpretation and critically reviewed the manuscript. SB assisted in interpretation of data and drafting and critically reviewed the manuscript. All authors read and approved the final manuscript.

## Pre-publication history

The pre-publication history for this paper can be accessed here:

http://www.biomedcentral.com/1471-2431/14/49/prepub
